# 

**Published:** 1875-06

**Authors:** 


### Bibliographical.

Pneumo-Thorax. By Austin Flint, Sr., M.D. Being one of the series of American Clinical Lectures edited by E. C. Seguin, M.D. New York: G. P. Putnam's Sons. 1875. Octavo, paper, pp. 19. Price 40 cents.

---

A Case of Reflex Neuralgia, associated with urethral contractions and a rare form of urinary sinus, with a description of the Cold Water Coil. By Fessenden N. Otis, M.D. New York: D. Appleton & Co. 1875. Pamphlet, pp. 12.

RUPTURE OF THE PERINEUM, with a description of a new operation. By D. Warren Brickell, M.D., Professor of Obstetrics and Diseases of Women in Charity Hospital Medical College, New Orleans. Pamphlet, pp. 9.

---

ICHTHYOSIS OF THE TONGUE AND VULVA. By Robert F. Weir, M.D., Surgeon Rosevelt Hospital. New York: Pamphlet, pp. 19.

---

PHYSIOLOGICAL ACTION OF THEBAIN. By J. Ott, M.D, of Easton, Penn. Pamphlet, pp. 12.

---

DUGAS' PATHOGNOMONIC SIGN OF DISLOCATIONS OF THE SHOULDER-JOINT. By W. T. Briggs, M.D. Nashville, Tenn. Pamphlet, pp. 9.

---

PROPOSTA INTORNO LA CURA DELLA LISSA DETTA COMMUNEMENTE RABBIA CANINA O IDROFOBIA. ROMA. 1875. Pamphlet, pp. 32.

---

ANALYSIS OF ONE THOUSAND CASES OF SKIN DISEASE, with cases and remarks on treatment. By L. Duncan Bulkley, A.M., M.D., of New York. Louisville: 1875. Pamphlet, pp. 29.

---

THE PRESENT STATUS OF ELECTRICITY IN MEDICINE, being the Semi-Annual Address before the Rhode Island Medical Society. By William F. Hutchinson, A.M., M.D. Providence. 1875. Pamphlet, pp. 29.

---

A DESCRIPTION OF NEW INSTRUMENTS for making examinations and applications to the cavities of the nose, throat and ear, and some remarks about the local and general treatment of the affections in which they are applicable. By Thomas F. Rumbold, M.D. Pamphlet, pp. 24.

---

AN ADDRESS ON THE CLIMATOLOGY OF FLORIDA, delivered before the Medical Association of the State of Florida. By A. S. Baldwin, M.D., President. Pamphlet, pp. 39.

---

VALEDICTORY ADDRESS to the Medical Graduates of the University of Louisville: March 1st, 1875. By David W. Yandell, M.D. Pamphlet, pp. 21.

Fifth Report of the New York Ophthalmic and Aural Institute. Pamphlet, pp. 16.

---

Eleventh Annual Report of the Alumni Association of the Philadelphia College of Pharmacy. 1875. Pamphlet, pp. 32.

---

Annual Report of the Managers of the Western Pennsylvania Hospital for 1874. Pamphlet, pp. 86.

